# Decreased Pulmonary Function in School Children in Western Japan after Exposures to Asian Desert Dusts and Its Association with Interleukin-8

**DOI:** 10.1155/2015/583293

**Published:** 2015-04-28

**Authors:** Masanari Watanabe, Hisashi Noma, Jun Kurai, Hiroyuki Sano, Rumiko Saito, Satoshi Abe, Yutaka Kimura, Setsuya Aiba, Mitsuo Oshimura, Akira Yamasaki, Eiji Shimizu

**Affiliations:** ^1^Department of Respiratory Medicine and Rheumatology, Tottori University Hospital, Yonago 683-8504, Japan; ^2^Department of Data Science, Institute of Statistical Mathematics, Tokyo 190-8562, Japan; ^3^Department of Respiratory Medicine and Allergology, Kinki University Faculty of Medicine, Osakasayama 589-8511, Japan; ^4^Department of Integrative Genomics, Tohoku Medical Megabank Organization, Tohoku University, Sendai 980-8574, Japan; ^5^Chromosome Engineering Research Center, Tottori University, Tottori 683-8504, Japan; ^6^Department of Dermatology, Tohoku University Graduate School of Medicine, Sendai 980-8574, Japan

## Abstract

The objective of the study was to investigate the influence of Asian dust storms (ADS) on pulmonary function of school children and the relationship of this effect with interleukin-8. Morning peak expiratory flow (PEF) was measured daily in 399 children from April to May 2012 and in 384 of these children from March to May 2013. The data were analyzed for an association between ADS events and PEF by linear mixed models. Interleukin-8 transcriptional activity was assessed in THP-G8 cells stimulated by airborne particles collected on ADS days. Seven ADS days were identified: April 23 and 24, 2012; March 8 to 10, 2013; and March 19 and 20, 2013. Changes in PEF after ADS exposure were −8.17 L/min (95% confidence interval, −11.40 to −4.93) in 2012 and −1.17 L/min (−4.07 to 1.74) in 2013, and there was a significant difference between 2012 and 2013. Interleukin-8 transcriptional activity was significantly higher in 2012 at 10.6 ± 2.9-fold compared to 3.7 ± 0.4 in March 8 to 10, 2013, and 2.3 ± 0.2 in March 19 and 20, 2013. The influence of ADS events on pulmonary function of children differs with each ADS event and may be related to interleukin-8 production.

## 1. Introduction

Asian dust storms (ADS) originating in the deserts of Mongolia, Northern China, and Kazakhstan often disperses dust over East Asia from spring until late autumn and is the second strongest source of dust emission worldwide [1]. An ADS is also a source of air pollutants because the dust contains chemicals, contaminating metals, microorganisms, and ionic components [2–4]. Therefore, ADS is a serious health problem associated with heavy pollution.

Numerous epidemiologic studies have shown that exposure to ADS increases rates of mortality, emergency treatment, and hospitalization for cardiovascular disease and pulmonary disease [5–8]. Other studies have shown that ADS increases the risk of hospitalization and exacerbates pulmonary function and respiratory symptoms in patients with asthma in Japan and South Korea [9, 10]. However, some studies from Taiwan have suggested that there is no significant association of ADS with asthma [11, 12]. Therefore, the influence of ADS on asthma may differ in different regions. This may be associated with differences in the materials attached to ADS airborne particles, which is influenced by the path the particles take [2–4]. We have also shown that effects on lower respiratory tract symptoms in adult patients with asthma differ for each ADS event [13]. Onishi et al. suggested that ADS events can be classified into three types based on Lidar data: Type 1 events with high counts of air pollution aerosols, Type 2 events with high counts of mineral dust particles, in comparison to air pollution aerosols, and Type 3 events with very low counts of air pollution aerosols [4].

Neutrophils migrate to the lung during acute inflammation induced by exposure to air pollutants [14]. The concentration of interleukin-8 (IL-8) in bronchial lavage fluid and IL-8 mRNA expression in bronchial biopsy tissue from healthy subjects are also increased by air pollutants [15]. IL-8 is increased in the blood of asthma patients during exacerbation [16] and thus is thought to be a key cytokine in exacerbation of asthma. In this context, we found that airborne particles collected on ADS days in Western Japan induced production of IL-8 in THP-G8 cells, whereas this effect did not occur with the original soil of the ADS [17].

In 2012, a study was already conducted to investigate the influence of ADS and air pollutants on pulmonary function of school children in Western Japan. In the current study, to investigate the difference of the influence of ADS events on pulmonary function in children, we conducted an extended survey in 2013, which was to monitor daily peak expiratory flow (PEF) in the same children as the 2012 investigation. Each year, using an IL-8 luciferase assay, these related detrimental effects on pulmonary function and differences in IL-8 promoter activity induced by ADS airborne particles were studied.

## 2. Materials and Methods

### 2.1. Subjects

The aim of this longitudinal follow-up study was to examine effects of ADS events on pulmonary function in school children. Daily morning PEF of children was monitored from March to May 2012 and 2013 because ADS events are most frequent in these months. March 2012 was used as trial period to allow the children to familiarize themselves with the monitoring. There was no ADS event in March 2012. The study was performed in Matsue, the capital city of Shimane Prefecture, in Southwest Japan. The population of Matsue is about 200,000 and the area is 530.2 km². In March 2012, all 401 fourth grade students aged 8 to 9 years from 4 of 35 elementary schools in Matsue were enrolled in the study. The four elementary schools were within 10 km of each other and all subjects lived within a radius of 1 km of the schools.

**Figure 1:**
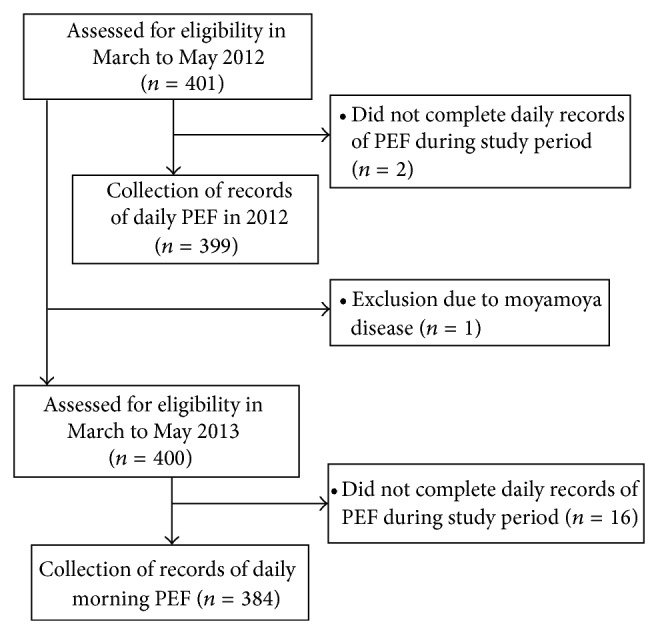
Flow chart showing the disposition of children in the study.

The disposition of the children in the study is shown in Figure 1. A total of 401 children were recruited into the study in March 2012. Two were subsequently excluded due to failure to keep a daily record for PEF. Thus, records of daily PEF were analyzed for 399 children in 2012. In March 2013, we recruited the same 401 children, of whom one was excluded due to Moyamoya disease. Sixteen children were subsequently excluded due to failure to keep a daily record for PEF. Thus, records of daily PEF were analyzed for 384 children in 2013.

The subjects recorded their age, gender, height, weight, and presence of asthma, allergic rhinitis, allergic conjunctivitis, atopic dermatitis, and food allergy in March 2012 and March 2013. Subjects were defined as having asthma if they met any of the following criteria in the past 12 months: (1) diagnosis of asthma by a pediatrician, (2) wheezing, (3) use of asthma medication, and (4) visiting a hospital regularly for asthma. Similarly, allergic rhinitis, allergic conjunctivitis, atopic dermatitis, and food allergy were judged to be present if the subjects met any of the following criteria in the past year: (1) diagnosis by a pediatrician, (2) use of medication for the disease, and (3) visiting a hospital regularly for the disease.

The study was approved by the institutional ethics committee (Ethics Committee of Tottori University, approval number 1764). We asked the Matsue City Board of Education for their help and received approval to submit the study to the schools. The study was also approved by the Parent Teacher Association (PTA) of each elementary school. Children and their parents were informed by teachers and we obtained formal consent for the study from the Matsue City Board of Education.

### 2.2. Monitoring of PEF

Before the study, the children and teachers were taught how to measure PEF. All children then measured their morning PEF daily using a peak flow meter (Mini-Wright, Harlow, England, American Thoracic Society Scale) from March to May 2012 and 2013, except for weekends and public holidays. Children recorded their best PEF value from three attempts after arriving at school between 8 a.m. and 9 a.m.

### 2.3. Definition of ADS Days and Air Pollutant Monitoring

The Japan Meteorological Agency has observatories throughout Japan and defines an ADS day based on a criterion of visibility <10 km due to dust arising from the deserts of East Asia, as determined by meteorological satellites monitoring each area. In this study, we used data from the Matsue observatory and we also referred to data from Light Detection and Ranging (Lidar) to define an ADS day. Lidar depolarization measurements performed simultaneously at two wavelengths can be used to identify nonspherical dust particles, which are mineral dust particles, and spherical aerosols such as organic aerosols and inorganic sulfates and nitrates [19, 20]. Thus, Lidar can be used to measure levels of mineral dust particles as airborne sand dust particles and nonmineral dust particles as air pollution aerosols in real time. Lidar data are collected continuously in 23 locations in Japan, South Korea, China, Mongolia, and Thailand to detect a potential ADS. Other studies have defined ADS events as a daily (24-hour) average of mineral dust particles >0.1 km^−1^ [9] or 0.066 km^−1^ (moderate ADS day) and 0.105 km^−1^ (heavy ADS day) [21].

### 2.4. Preparation of Airborne Particles Collected on ADS Days

On ADS days, dust particles were collected at Tottori Prefectural Institute of Health and Environment, Yurihama, Tottori, which is located 70 km east of Matsue. This is the most suitable area close to Matsue to monitor particulate matter from East Asia because Yurihama is rural and has no source of air pollutants, except for motor vehicles. The observatory in Yurihama is also located away from populated areas. ADS airborne particles were collected in Tottori on April 23 and April 24, 2012; March 8 to 10, 2013; and March 19 and 20, 2013, using a high-volume air sampler (HV-1000R, Shibata, Tokyo, Japan) on the roof of a building. ADS airborne particles were filtered based on their aerodynamic diameters (Andersen Sampler, Shibata) into 5 sizes (<1.1, 1.1–2.0, 2.0–3.3, 3.3–7.0, and >7.0 *μ*m) and each filter was dried in a desiccator before and after sampling to be weighed. ADS airborne particles of 3.3–7.0 *μ*m were subsequently used in the study. Collected airborne dust was sterilized at 121°C for 30 min in an autoclave (Tomy SX-300, Tomy, Tokyo, Japan) to prevent growth of bacteria and fungi and dried at 80°C for 4 h with drying sterilizer (SG600, Yamato Scientific, Tokyo, Japan). The collected airborne dust was then weighed and stored in a freezer at −20°C. Soil from the China Loess Plateau (CJ-1), the original ADS soil from the Tengger Desert and Huining County located in Gansu Province, was obtained from the National Institute for Environmental Studies (Ibaraki, Japan) in 2002. This reference material is certified by the National Institute for Environmental Studies and the National Research Center for Environmental Analysis and Measurement (Beijing, China). To stimulate THP-G8 cells, airborne particles collected on ADS days were diluted to 1 mg/mL with distilled deionized water.

### 2.5. IL-8 Promoter-Luciferase Gene Reporter Assay and Measurements of IL-8 and Endotoxin

THP-G8 cells are a THP-1-derived reporter cell line that express stable luciferase orange (SLO) and stable luciferase red (SLR) genes under control of the IL-8 and glyceraldehyde 3-phosphate dehydrogenase (GAPDH) promoters, respectively [22]. The THP-G8 cell line was kindly provided by the Department of Dermatology, Tohoku University Graduate School of Medicine, Sendai, Japan, and was cultured as described previously [22]. We first stimulated THP-G8 cells (5 × 10^4^ cells/100 *μ*L/well) in 96-well black plates (Greiner Bio-One GmbH, Frickenhausen, Germany) with lipopolysaccharide (LPS) (Wako Pure Chemicals, Osaka, Japan) and examined IL-8 and GAPDH reporter activity for various time periods and concentrations. Luciferase activity was determined using a microplate luminometer with a Phelios multicolor detection system (Atto, Tokyo, Japan) using Tripluc luciferase assay reagent (Toyobo, Osaka, Japan). IL-8 transcriptional activity was assessed from normalized SLO luciferase activity (nSLO-LA), which was calculated as SLO-LA divided by SLR-LA, and the fold induction of nSLO-LA was calculated as the nSLO-LA level of treated cells divided by that of untreated cells [22]. Induction of IL-8 transcriptional activity was measured after THP-G8 cells were stimulated for 5 h with solvent only (negative control), 100 ng/mL lipopolysaccharide (LPS), and ADS airborne particles collected in 2012 and 2013.

IL-8 concentrations in culture supernatants were determined using an enzyme-linked immunosorbent assay (ELISA) kit for IL-8 (R&D Systems, Minneapolis, MN, USA). Samples were run in triplicate and read using an automated ELISA reader (Model 680, Bio-Rad, Philadelphia, PA, USA). The range of the assay was 31.2 to 2000 pg/mL. Endotoxin concentrations in ADS airborne particles were measured using a chromogenic LAL endotoxin assay kit (GenScript, Piscataway, NJ, USA). The range of the assay was 0.01 to 1 EU/mL. The pH of ADS airborne particles was measured with a pH meter (MP220, Mettler Toledo, Schwerzenbach, Switzerland).

### 2.6. Measurement of Metal Elements in CJ-1 Soil and ADS Airborne Particles

Metal elements in CJ-1 soil and ADS airborne particles collected on April 23 and 24, 2012; March 8 to 10, 2013; and March 19 and 20, 2013 were measured by Oki Engineering (Tokyo, Japan). The concentrations of aluminum (Al), arsenic (As), barium (Ba), calcium (Ca), cadmium (Cd), cobalt (Co), chromium (Cr), copper (Cu), iron (Fe), mercury (Hg), potassium (K), lanthanum (La), magnesium (Mg), manganese (Mn), sodium (Na), nickel (Ni), phosphorus (P), lead (Pb), strontium (Sr), titanium (Ti), and zinc (Zn) were measured by inductively coupled plasma atomic emission spectrometry. Silicon (Si) was measured using electrothermal atomic absorption spectrometry.

### 2.7. Statistical Analysis

To evaluate the effects of exposure to an ADS on the daily PEF of children, linear mixed models that accounted for correlations among repeated measurements within a subject were used to estimate the effects of exposure to an ADS on the daily PEF of children in April to May 2012 and March to May 2013 [23, 24]. Additionally, to adjust for potential confounding factors, we used linear mixed models with the following general form:

(1) $$\begin{array}{c} Y_{ij}=\beta_{0}+\beta_{1}x_{1,j}+\overset{p}{\underset{k=1}{\sum }}\beta_{k}x_{k,ij}+b_{0,i}+\varepsilon_{ij}. \end{array}$$

*Y*_*ij*_ corresponds to the daily PEF for the *i*th child at the* j*th day (*i* = 1,2,…, *N*; *j* = 1,2,…, *T*). *x*_1,*j*_ is an exposure variable of* j*th day (measurement of air pollution), and *x*_*k*,*ij*_  (*k* = 2, 3,…, *p*) are potential confounding factors involving individual characteristics (age, gender, height, weight, and presence of asthma, allergic rhinitis, allergic conjunctivitis, atopic dermatitis, and food allergies) and meteorological variables such as daily temperature, humidity, and atmospheric pressure. *β*_0_, *β*_1_,…, *β*_*p*_ are corresponding fixed effects coefficients, and *b*_0,*i*_ is the random effect of intercept for *i*th child and are assumed to be *b*_0,*i*_ ~ *N*(0, *σ*_*b*_^2^). *ε*_*ij*_ is the error term, *ε*_*ij*_ ~ *N*(0, *σ* ^2^). In addition, effects on PEF were measured from the day of ADS exposure until 3 days after exposure because a dust effect on PEF can persist for up to 3 days [10]. Differences in PEF between the 2012 and 2013 results were also evaluated. The two-pollutant model was applied to different combinations of pollutants to assess the stability of the effects of ADS on PEF after adjustment for individual characteristics (age, gender, height, weight, and presence of asthma, allergic rhinitis, allergic conjunctivitis, atopic dermatitis, and food allergies) and meteorological variables (temperature, humidity, and atmospheric pressure). R version 3.0.3 (R Foundation for Statistical Computing, Vienna, Austria) was used for statistical analysis of PEF values and ADS exposure. Differences of nSLO-LA of THP-G8 cells were analyzed by ANOVA using SPSS Statistics (Japanese version 21.0 for Windows, IBM Japan, Tokyo, Japan). All quoted *P* values are two-sided and the significance level was set to 0.05.

## 3. Results

### 3.1. Profile of the Children

The characteristics of the children in the 2012 and 2013 studies are shown in Table 1.

### 3.2. Air Pollution Levels and Weather Information on ADS Days and Non-ADS Days

**Figure 2:**
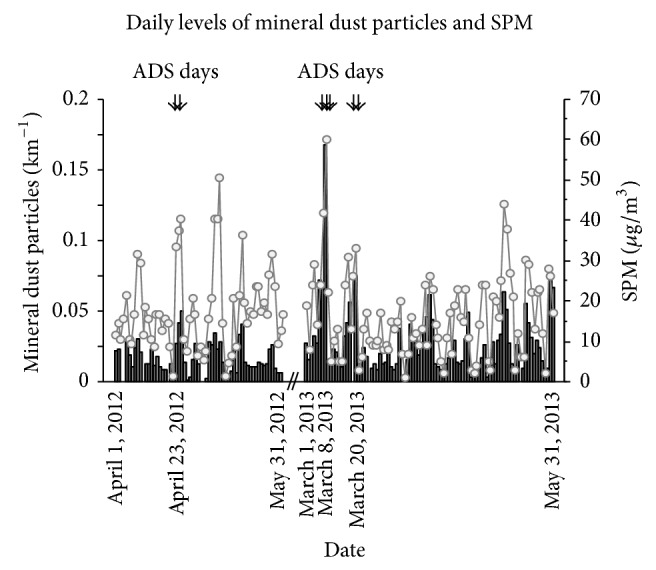
Daily levels of mineral dust particles (airborne sand dust particles) (bar graph) and SPM (line graph). Arrows indicate ADS days.

In 2012, April 23 and 24 were identified as ADS days. In 2013, March 8 to 10 and 19 and 20 were similarly identified as ADS days. Non-ADS days were defined as all other days from April 1 to May 31, 2012, and from March 1 to May 31, 2013. Daily levels of mineral dust particles (airborne sand dust particles) and suspended particulate matter (SPM) are shown in each period in Figure 2. The levels of air pollutants and weather during the study periods are shown in Table 2.

### 3.3. PEF

**Figure 3:**
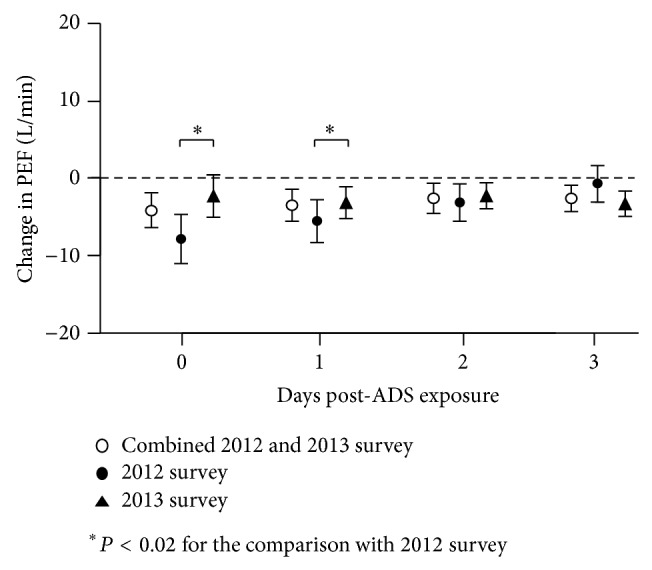
PEF changes caused by an ADS event from 0 (ADS day) to 3 days after ADS exposure in combined 2012 and 2013 (open circles), 2012 (black circles), and 2013 (triangles), with 95% confidence intervals (error bars). Data are controlled for age, gender, height, weight, and presence of asthma, allergic rhinitis, allergic conjunctivitis, atopic dermatitis, and food allergies; meteorological variables such as daily temperature, humidity, and atmospheric pressure; and the linear time trend. There are significant differences in the decrement of PEF on days 0 and 1 between 2012 and 2013 (^*^ *P* < 0.02).

Changes in PEF after exposure to ADS are shown in Figure 3. In order to show the post-ADS-exposure effects, these changes are shown from 0 (ADS day) to 3 days after ADS exposure. In combining 2012 and 2013, the changes in PEF after exposure to ADS exposure were −4.16 L/min (95% CI, −6.33 to −1.99) on day 0, −2.97 L/min (−5.03 to −0.91) on day 1, −2.23 L/min (−4.12 to −0.35) on day 2, and −2.57 L/min (−4.29 to −0.86) on day 3 after ADS. There were significant decreases in PEF from day 0 to day 3 after ADS exposure. In 2012, the changes in PEF were −7.82 L/min (−10.93 to −4.71) on day 0, −5.49 L/min (−8.14 to −2.85) on day 1, −3.15 L/min (−5.54 to −0.75) on day 2, and −0.72 L/min (−3.03 to 1.59) on day 3 after ADS. A significant decrease in PEF persisted for 2 days after ADS exposure in 2012. In 2013, the changes in PEF were −2.33 L/min (−5.09 to 0.44) on day 0, −2.72 L/min (−4.80 to −0.64) on day 1, −2.26 L/min (−3.98 to −0.53) on day 2, and −3.04 L/min (−4.68 to −1.40) on day 3 after ADS. A significant decrease in PEF continued from days 1 to 3 after ADS exposure. On days 0 and 1, the decrease in PEF after exposure to ADS in 2012 was significantly higher than that in 2013. In addition, the 2012 and 2013 forest plots indicate clear differences. Significant differences were observed in PEF on days 0 and 1, and the decrement of PEF in 2012 was higher than that in 2013. In a two-pollutant model adjusted for SPM, PM_2.5_, SO_2_, NO_2_, and O_*x*_, an ADS event in 2012 alone was significantly associated with a decrease of PEF (Table 3). In contrast, in 2013, a similar model gave no significant relationship between ADS events and PEF in children.

### 3.4. IL-8 Transcriptional Activity and IL-8 Secretion in THP-G8 Cells

**Figure 4:**
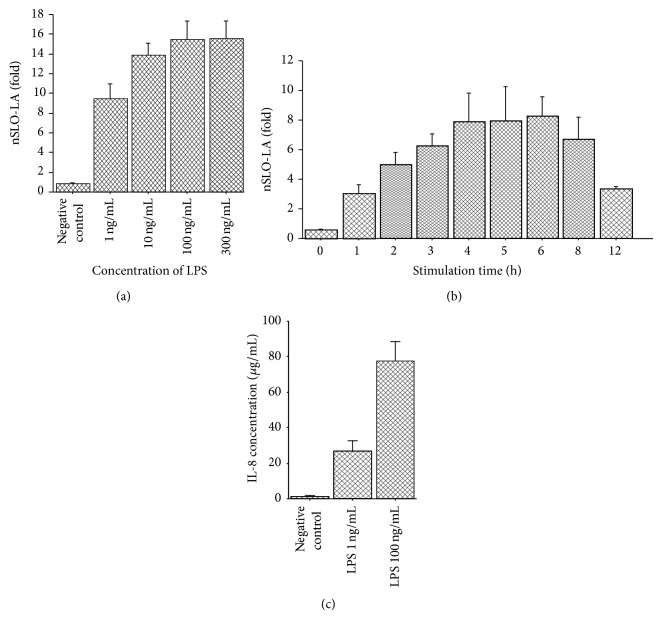
(a) IL-8 transcriptional activity in THP-G8 cells stimulated with LPS at various concentrations (*n* = 6) for 5 h. IL-8 transcriptional activity is based on normalized SLO luciferase activity (nSLO-LA), which was calculated as SLO-LA divided by SLR-LA. The fold induction of nSLO-LA was calculated as the nSLO-LA of treated cells divided by that of untreated cells [18]. (b) IL-8 transcriptional activity in THP-G8 cells stimulated with 100 ng/mL LPS (*n* = 6) for various time periods. (c) Concentrations of IL-8 in supernatants of a stable THP-1-derived IL-8 reporter cell line stimulated with solvent only (negative control), LPS (*n* = 6, 1 ng/mL), and LPS (*n* = 6, 100 ng/mL). The IL-8 concentration was measured using an ELISA kit. Samples were run in triplicate. The assay range was 31.2 to 2000 pg/mL.

In THP-G8 cells stimulated for 5 h with various LPS concentrations, nSLO-LA (a measure of IL-8 transcriptional activity) reached a plateau at 100 ng/mL LPS (Figure 4(a)). Maximum induction of nSLO-LA by LPS (100 ng/mL) occurred between 4 and 6 h (Figure 4(b)). Based on these results, we subsequently used stimulation for 5 h to investigate the effect of ADS airborne particles on IL-8 transcriptional activity. The concentrations of IL-8 in supernatants of THP-G8 cells stimulated with vehicle, LPS (*n* = 6, 1 ng/mL), and LPS (*n* = 6, 100 ng/mL) were 1.2 ± 0.2, 26.6 ± 6.2, and 77.4 ± 10.9 *μ*g/mL, respectively (Figure 4(c)). This increase in IL-8 secretion is in agreement with the augmentation of nSLO in THP-G8 cells.

**Figure 5:**
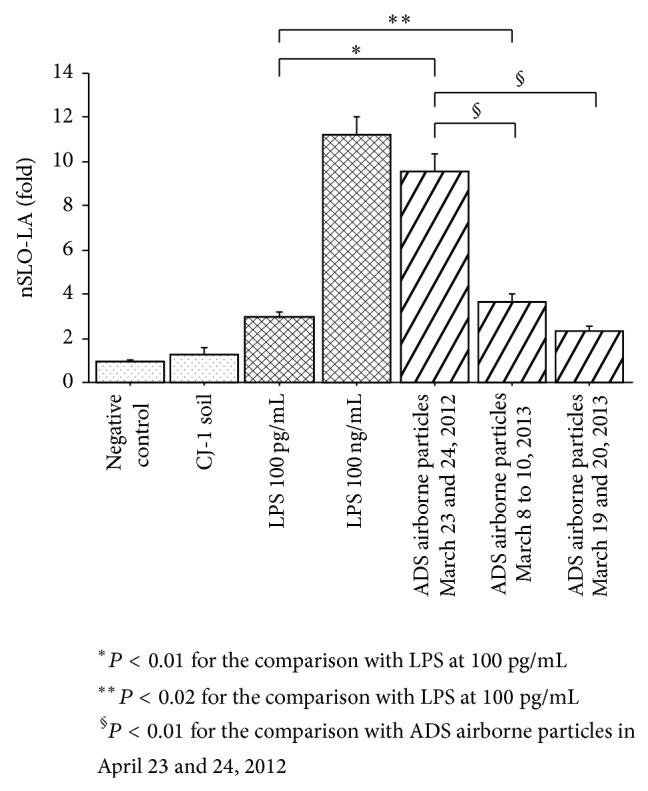
IL-8 transcriptional activity measured using an IL-8 luciferase assay in a stable THP-1-derived IL-8 reporter cell line. Cells were treated with solvent only (*n* = 6, negative control), LPS (*n* = 6, 100 pg/mL, positive control), LPS (*n* = 6, 100 ng/mL, positive control), and ADS airborne particles collected on April 23 and 24, 2012 (*n* = 6, 1 mg/mL), March 8 to 10, 2013 (*n* = 6, 1 mg/mL), and March 19 and 20, 2013 (*n* = 6, 1 mg/mL). ^*^ *P* < 0.01 versus LPS at 100 pg/mL, ^**^ *P* < 0.02 versus LPS at 100 pg/mL, and ^§^ *P* < 0.01 versus ADS airborne particles from April 23 to April 24, 2012.

The pH values of ADS airborne particles (1 mg/mL) collected on April 23 and 24, 2012; March 8 to 10, 2013; and March 19 and 20, 2013 were 7.9, 7.6, and 7.6, respectively. The nSLO-LA values (IL-8 transcriptional activity) of THP-G8 cells (Figure 5) changed by 0.95 ± 0.09-fold (vehicle, *n* = 6), 2.87 ± 0.28-fold (LPS, *n* = 6, 100 pg/mL), 11.21 ± 0.28-fold (LPS, *n* = 6, 100 ng/mL), 9.56 ± 0.80-fold (ADS particles from April 23 to 24, 2012, *n* = 6, 1 mg/mL), 3.65 ± 0.36-fold (ADS particles from March 8 to 10, 2013, *n* = 6, 1 mg/mL), and 2.33 ± 0.24-fold (ADS particles from March 19 to 20, 2013, *n* = 6, 1 mg/mL).

The pH value of CJ-1 soil was constant at 8.4 for each event. The pH values of ADS airborne particles (1 mg/mL) collected on April 23 and 24, 2012; March 8 to 10, 2013; and March 19 and 20, 2013 were 7.9, 7.6, and 7.6, respectively. THP-G8 cells were stimulated with CJ-1 soil after adjusting the pH of the soil to 7.8 with 0.1 N sodium hydroxide. The nSLO-LA values (IL-8 transcriptional activity) of THP-G8 cells (Figure 5) changed by 0.95 ± 0.09-fold (vehicle, *n* = 6), 1.48 ± 0.27-fold (CJ-1 soil, *n* = 6, 1 mg/mL), 2.87 ± 0.28-fold (LPS, *n* = 6, 100 pg/mL), 11.21 ± 0.28-fold (LPS, *n* = 6, 100 ng/mL), 9.56 ± 0.80-fold (ADS particles from April 23 to 24, 2012, *n* = 6, 1 mg/mL), 3.65 ± 0.36-fold (ADS particles from March 8 to 10, 2013, *n* = 6, 1 mg/mL), and 2.33 ± 0.24-fold (ADS particles from March 19 to 20, 2013, *n* = 6, 1 mg/mL). nSLO-LA values in THP-G8 cells stimulated by ADS airborne particles differed significantly from those of controls and cells stimulated with 100 pg/mL LPS. nSLO-LA values also differed significantly for each pairwise comparison of airborne particles collected in the three ADS periods.

### 3.5. Endotoxin Concentration in Airborne Particles Collected on ADS Days

The endotoxin levels in ADS airborne particles (1 mg/mL) collected on April 23 and 24, 2012; March 8 to 10, 2013; and March 19 and 20, 2013 were 0.19, 0.08, and 0.07 EU/mL, respectively. These values were all lower than the level of 0.89 EU/mL found in 100 pg/mL LPS. The endotoxin concentration in 100 ng/mL LPS was out of the range of the assay.

### 3.6. Concentration of Metal Elements in CJ-1 Soil and Airborne Particles Collected on ADS Days

The concentrations of metal elements in CJ-1 soil and ADS airborne particles collected on April 23 and 24, 2012; March 8 to 10, 2013; and March 19 and 20, 2013 are shown in Table 4.

## 4. Discussion

To investigate the effect of ADS on pulmonary function, we monitored daily PEF in school children from April to May 2012 and March to May 2013 and found a significant correlation between exposure to an ADS and pulmonary function. The 2012 survey alone also showed this relationship. When differences in PEF between the 2012 and 2013 results were evaluated, the same relationship of PEF with ADS events was not found in 2013, despite the study being conducted in the same children. The decline of PEF upon ADS exposure in 2012 was also significantly higher than that in 2013, and IL-8 transcriptional activity in THP-G8 cells induced by ADS airborne particles collected in 2012 was also significantly higher than that induced by ADS airborne particles collected in 2013. These results suggest that the effect of ADS on pulmonary function in children is associated with enhanced airway inflammation mediated by elevation of IL-8.

Desert sand can reduce pulmonary function in patients with asthma after exposure at a level of PM_10_ ranging from 1500 to 2000 *μ*g/m^3^/hour [25]. This level of PM_10_ is 10 to 20 times higher than that during an ADS event in Japan. In the current study, the level of mineral dust particles on ADS events on March 8 to 10, 2013, and March 19 and 20, 2013 was about twice as high as that in 2012. However, the decrease of PEF after exposure to ADS in 2012 was higher than that in 2013. These results suggest that the decline in pulmonary function of school children during an ADS event has little connection to mineral dust particles (sand dust particles). Nonmineral dust particles were similar on ADS events in 2012 and 2013. ADS events in 2012 had higher counts of air pollution aerosols than those in 2013. Based on Onishi's criteria [4], the ADS event in 2012 can be classified as Type 1 and those in 2013 as type 2. The air pollution aerosols during ADS can be considered a cause of the significant difference in the effect of the ADS on pulmonary function of school children between 2012 and 2013.

The ADS days in 2013 were defined as moderate, while, on April 23 and 24, 2012, the mean daily average concentration of mineral dust particles was 0.046 ± 0.006 km^−1^ in Matsue City. These values are lower than the thresholds defined in previous studies [9, 21]. However, the daily average level of mineral dust particles on ADS days was higher than that on non-ADS days, as required for definition of an ADS day in the Japan Meteorological Agency criteria used in this study.

Many studies have shown that children are susceptible to air pollution such as NO_2_, O_*x*_, and SO_2_ [18, 26]. Therefore, we used a linear mixed model and a two-pollutant model to adjust for the effects of NO_2_, O_*x*_, and SO_2_ on pulmonary function. In both models, ADS in 2012 remained significant after inclusion of NO_2_, O_*x*_, and SO_2_. However, in the 2013 survey, we were not able to find a significant association with ADS and pulmonary function. These results suggest that airborne particles during ADS decrease pulmonary function irrespective of NO_2_, O_*x*_, and SO_2_.

IL-8 is a key cytokine in air pollutant-induced airway inflammation [15, 16]. Components that adhered to ADS particles can increase release of IL-6 and IL-8 from airway epithelial cells [27]. We showed that ADS airborne particles promote transcriptional activity and production of IL-8 in THP-G8 cells, with a significant increase in IL-8 transcriptional activity in THP-G8 cells treated with ADS airborne particles compared to those treated with original ADS soil (CJ-1 soil). Additionally, we measured the difference in production of IL-8 by particles collected during each ADS event. The IL-8 transcriptional activity of ADS airborne particles collected in 2012 was significantly higher than that for particles collected in 2013. This difference in production of IL-8 by ADS airborne particles may account for the different effects on pulmonary function in school children in 2012 and 2013.

The production of IL-8 induced by ADS airborne particles in 2012 had a significant difference compared to 2013. However, there was no difference between the two ADS airborne particles in 2013. The ADS events in 2013 had happened close together, and we suspect that the route and composition of the ADS airborne particles in the two events were similar. In fact, when we analyzed the effect on PEF between two ADS events in 2013 separately, the decreases in PEF after exposure to ADS were −4.1 L/min (95% CI, −10.6 to 2.4, *P* = 0.21) in March 8 to 10 and −3.3 L/min (95%CI, −10.8 to 4.1, *P* = 0.37) in March 19 and 20. In both ADS events in 2013, there was not a significant decrease of the effects on PEF. Therefore, we presented the combined results in the main analysis. The differences in the substances and the levels of those substances attached to desert sand dusts depend on the route along which desert sand dusts pass and may play an important role in the effect of ADS on pulmonary function in children.

According to the analysis of the concentrations of metal elements, ADS airborne particles in 2012 had more Al, Cu, Fe, K, and Ti compared to those on March 8 to 10 and March 19 and 20, 2013. The amounts of Al, Fe, and Ti in ADS airborne particles in 2012 were lower than CJ-1. Cu and K may play a causative role in the difference of production of IL-8 induced by ADS airborne particles. However, Kumar et al. indicated that Cu was not a cause of the production of CXCL1 (a mouse functional homologue of IL-8) and IL-6 induced by ambient and traffic-derived particulate matter, but it did indicate that Fe content of airborne particulate matter may be more important in mouse airway epithelial injury [28]. Metal components attached to ADS airborne particles may be one of the causes of the difference between 2012 and 2013, but further study is needed to determine a role of metal components attached to ADS on the effect of production of proinflammatory cytokines.

Ogino et al. found that some proteins contained in ambient particulate matter are important environmental factors that aggravate airway hyperresponsiveness and airway inflammation in mice [29]. An ADS contains different amounts of *β*-glucan, which can induce airway inflammation [30, 31]. Thus, in addition to chemical substances, anthropogenic metal components, and sulfate, some proteins and *β*-glucan attached to ADS airborne particles may play important roles in the reduction of pulmonary function during ADS events.

Inhaled LPS is associated with airway neutrophil inflammation in patients with asthma and in healthy subjects [32–34]. Our results show that ADS airborne particles contain endotoxin, and the endotoxin concentration of ADS airborne particle was lower than that in LPS at 100 pg/mL. However, the IL-8 transcriptional activity induced by ADS airborne particle collected on April 23 and 24, 2012 and March 8 to 10, 2013 was significantly higher than that induced by LPS at 100 pg/mL. Endotoxin may augment IL-8 transcriptional activity in THP-G8 cells, in addition to other substances on ADS airborne particles that may induce IL-8.

Park et al. [10] and Yoo et al. [35] found a relationship between ADS events and PEF in Korean children with asthma, while Hong et al. did not find a significant relationship between ADS events and PEF in children without asthma [36]. Patients with allergic diseases may also be more sensitive to air pollution [37–39]. Therefore, in this study, we analyzed the data after adjustment for allergic diseases. This analysis showed that there was a significant decrease of PEF on ADS days in 2012 compared to 2013, regardless of the presence of allergic diseases. However, the number of subjects with each disease was too small to investigate the association of PEF with ADS. Further studies are needed to define the relationship between ADS and PEF in children with allergic diseases.

In this study, children recorded their PEF value after arriving at school but did not record their PEF value on weekends and public holidays. The ADS days March 9, 10, and 20, 2013 lacked PEF data because they were holidays. However, this intermittent missing data is statistically independent of the ADS events. Thus, it would not cause any serious bias in the results. Although it would raise a reduction of statistical power, the significant associations were still observed in the primary analyses.

There are several limitations in the study. First, we did not investigate diseases other than asthma, allergic rhinitis, allergic conjunctivitis, atopic dermatitis, and food allergies. Second, we were unable to diagnose asthma based on airway hyperresponsiveness to methacholine and reversible airflow limitation. In this study, some children were considered to have asthma, when in fact their wheezing may have been caused by respiratory tract infection or other diseases. However, wheezing caused by respiratory tract infection and other diseases is more common in children under 6 years old, and that is younger than those in our study [40]. Additionally, it is difficult to distinguish asthma and reactive airway disease based on the present diagnostic criteria. Third, we were unable to measure the individual amount of exposure to ADS. Fourth, we did not analyze the composition of the ADS airborne particles. Therefore, this study was not able to investigate which components of ADS airborne particles played important roles in reduction of pulmonary function during the ADS and which components induce IL-8. Further studies are needed to define these components.

## 5. Conclusion

We conclude that the effect of exposure to ADS on pulmonary function in school children differed among ADS events, and that enhancement of IL-8 transcriptional activity also differed among ADS airborne particles collected during the respective events. These findings suggest that substances attached to ADS airborne particles exacerbate pulmonary function of school children. Further studies are needed to identify the substances attached to the ADS airborne particles that play key roles in exacerbation of pulmonary function.

## Figures and Tables

**Table 1:** Characteristics of children.

	2012	2013
Number	399	384
Gender (male/female)	205/194	194/190
Height (cm)	132.3 ± 5.9	137.7 ± 7.0
Male	132.2 ± 5.5	136.9 ± 6.3
Female	132.4 ± 6.4	138.5 ± 7.7
Weight (kg)	29.5 ± 5.8	32.4 ± 6.6
Male	29.6 ± 6.2	32.3 ± 6.8
Female	29.3 ± 5.4	32.6 ± 6.4
Allergic disease		
Asthma	38	45
Allergic rhinitis	78	74
Allergic conjunctivitis	8	15
Atopic dermatitis	44	36
Food allergy	19	20

Data are shown as the mean ± S.D.

**(a):** 

Measurement	ADS days		
	April 23 and 24, 2012	March 8 to 10, 2013	March 19 and 20, 2013
Daily average temperature, °C	17.6 ± 1.1	13.6 ± 3.8	12.4 ± 1.4
Daily maximum temperature, °C	23.3 ± 3.0	20.4 ± 0.9	18.6 ± 0.0
Daily minimum temperature, °C	13.1 ± 0.7	8.0 ± 5.8	7.9 ± 4.5
Daily average relative humidity, %	70.0 ± 5.7	64.7 ± 7.6	79.5 ± 3.5
Daily minimum relative humidity, %	48.0 ± 12.7	29.3 ± 10.7	52.0 ± 0.0
Daily average atmospheric pressure, hPa	1010.8 ± 1.0	1008.6 ± 3.1	1007.4 ± 3.0
Daily average mineral dust particles, km^−1^	0.046 ± 0.006	0.084 ± 0.077	0.075 ± 0.001
Daily average nonmineral dust particles, km^−1^	0.148 ± 0.097	0.138 ± 0.050	0.097 ± 0.001
Daily average SPM, *μ*g/m^3^	39.5 ± 2.1	44.3 ± 19.0	32.5 ± 5.0
Daily average PM_2.5_, *μ*g/m^3^	17.2 ± 1.3	37.3 ± 16.7	37.8 ± 4.5
Daily average SO_2_, ppb	1.3 ± 0.6	2.0 ± 1.1	1.9 ± 1.3
Daily average NO_2_, ppb	1.5 ± 0.4	3.4 ± 1.8	3.6 ± 0.6
Daily average O_*x*_, ppb	55.9 ± 5.7	63.1 ± 10.5	49.4 ± 5.3

**(b):** 

Measurement	Non-ADS days	
	2012	2013
Daily average temperature, °C	15.7 ± 3.5	13.2 ± 5.0
Daily maximum temperature, °C	20.9 ± 4.5	18.7 ± 5.7
Daily minimum temperature, °C	10.9 ± 4.0	8.1 ± 5.0
Daily average relative humidity, %	70.4 ± 9.3	69.8 ± 9.8
Daily minimum relative humidity, %	44.9 ± 15.4	42.8 ± 13.6
Daily average atmospheric pressure, hPa	1010.0 ± 5.7	1011.3 ± 5.9
Daily average mineral dust particles, km^−1^	0.016 ± 0.010	0.024 ± 0.017
Daily average non-mineral dust particles, km^−1^	0.042 ± 0.037	0.073 ± 0.049
Daily average SPM, *μ*g/m^3^	17.7 ± 10.1	17.8 ± 8.9
Daily average PM_2.5_, *μ*g/m^3^	10.3 ± 5.4	17.5 ± 7.3
Daily average SO_2_, ppb	0.9 ± 0.6	1.0 ± 0.8
Daily average NO_2_, ppb	2.6 ± 1.2	2.75 ± 1.2
Daily average O_*x*_, ppb	50.0 ± 7.5	50.2 ± 8.4

Data are presented as the mean ± S.D., Non-ADS days were all other days except for ADS days from April 1 to May 31, 2012, and March 1 to May 31, 2013.

**Table 3:** Estimated effects of ADS events on PEF in two-pollutant model after adjustment for SPM, PM_2.5_, NO_2_, O_*x*_, and SO_2_.

Year	Adjustment	Change in PEF	95% CI	*P* value
2012 and 2013	Adjusted for SPM	−3.00	−5.31, −0.68	0.011
	Adjusted for PM_2.5_	−3.60	−5.94, −1.27	0.002
	Adjusted for SO_2_	−2.14	−4.43,0.15	0.059
	Adjusted for O_*x*_	−3.49	−5.70, −1.28	0.002
	Adjusted for NO_2_	−4.20	−6.37, −2.03	0.001

2012	Adjusted for SPM	−6.04	−9.44, −2.64	0.001
	Adjusted for PM_2.5_	−6.48	−9.78, −3.18	0.001
	Adjusted for SO_2_	−7.41	−10.69, −4.13	0.001
	Adjusted for O_*x*_	−3.93	−7.25, −0.62	0.019
	Adjusted for NO_2_	−10.04	−13.42, −6.67	0.001

2013	Adjusted for SPM	−1.57	−4.56,1.43	0.306
	Adjusted for PM_2.5_	−1.97	−5.10,1.15	0.216
	Adjusted for SO_2_	−2.19	−5.02,0.63	0.128
	Adjusted for O_*x*_	0.19	−2.79,3.18	0.900
	Adjusted for NO_2_	−2.45	−4.38,1.49	0.085

Calculated for an interquartile by ADS and adjusted for individual characteristics (age, gender, height, weight, and presence of asthma, allergic rhinitis, allergic conjunctivitis, atopic dermatitis, and food allergies) and meteorological variables (temperature, humidity, and atmospheric pressure).

ADS: Asian dust storm, PEF: peak expiratory flow, SPM (*μ*g/m^3^): suspended particle matter, PM_2.5_ (*μ*g/m^3^): particulate matter smaller than 2.5 *μ*m in diameter, NO_2_ (ppb): nitrogen dioxide, O_*x*_ (ppb): photochemical oxidants, SO_2_ (ppb): sulfur dioxide, and CI: confidence interval.

**Table 4:** Concentration of metal elements in CJ-1 soil and airborne particles collected on ADS days.

Metals (*μ*g/mg)	CJ-1 soil	ADS airborne particles in April 23 and 24, 2012	ADS airborne particles in March 8 to 10, 2013	ADS airborne particles in March 19 and 20, 2013
Al	68.00	28.80	22.40	14.80
As	ND	ND	ND	ND
Ba	0.44	0.12	0.18	0.10
Ca	68.00	29.60	44.00	31.20
Cd	ND	ND	ND	ND
Co	0.01	ND	ND	ND
Cr	0.05	ND	ND	ND
Cu	0.03	0.12	ND	0.07
Fe	26.00	22.00	20.80	14.40
Hg	ND	ND	ND	ND
K	0.19	0.38	0.33	0.30
La	0.03	ND	ND	ND
Mg	18.00	14.00	16.80	14.40
Mn	0.70	0.52	0.56	0.40
Na	0.44	33.60	56.00	64.00
Ni	0.03	0.14	0.15	0.11
P	0.68	ND	ND	ND
Pb	0.02	0.06	0.12	0.06
Si	260.00	140.00	108.00	72.00
Sr	0.26	0.16	0.22	0.16
Ti	2.40	0.96	0.92	0.52
Zn	0.07	0.52	0.64	0.60

ADS: Asian dust storm, CJ-1 soil: soil from the China Loess Plateau, the original ADS soil in the Tengger Desert and Huining located in Gansu Province, and ND: not detected.
